# Society Meetings

**Published:** 1900-02

**Authors:** 


### SOCIETY MEETINGS.

THE Medical Society of the County of Erie at its seventy-ninth annual meeting, held January 9, 1900, presented a program of unusual merit. Besides the president's address on the water supply and disposal of sewage, comment upon which is made elsewhere, papers were presented by Dr. Chauncey P. Smith, on The importance of early diagnosis of tumors of the breast; by Dr. Maud J. Frye, on The neuroses of childhood; and by Dr. William G. Taylor, on Unusual condition of the skin and kidneys following an operation for appendicitis.

Dr. Smith demonstrated intimate knowledge of his subject, spoke extemporaneously and presented a large number of specimens of great interest, illustrating the several points that he accentuated—all of which made a good impression on his auditors.

Dr. Frye's paper was well written, and possessed the charm of much originality of thought, as well as ingenuity in form. The paper of Dr. Taylor was full of interest from the start, because of the novelty that attached to it, as well as the excellence of its manner of presentation.

This meeting demonstrates what can be done by well directed effort; it shows that this venerable society is full of youthful force; and it further serves notice on those who fail to attend its meetings that they, themselves, are the losers.

---

THE Niagara Falls Academy of Medicine held its last regular monthly meeting at the office of Dr. Frank Guillemont, 550 Main Street, Monday, January 8, 1900, at 8.45. Dr. Miller read a paper on the Obstetrical douche and the discussion was opened by Dr. Potter.

---

THE Medical Society of the State of New York is preparing to hold its ninety-fourth annual meeting just as this issue of the journal is being delivered to its subscribers. It is of the first importance that all the official delegates from Erie County shall attend this meeting in order to perfect their membership as their term will then expire, and new delegates will take their seats next year.

The most important feature of this meeting is perhaps, the discussion on tuberculosis, which is scheduled for Tuesday evening in the Assembly chamber. The following is the scheme of the debate:

State care of tuberculosis patients. (1) The duty of the state and municipality in the care of pulmonary tuberculosis among the poor, Edward O. Otis, Boston, Mass; (2) Remarks upon the work accomplished at the State Hospital for Consumptives, Rutland, Mass., Vincent Y. Bowditch, Boston, Mass; (3) Some results of the climatic and sanatorium treatment of tuberculosis in the Adirondacks, Edward R. Baldwin, Saranac Lake; (4) Infectious character of tuberculosis and the prognosis of incipient pulmonary consumption, George Blumer, Albany; (5) The policy of the state relative to the spread of tuberculosis, Enoch V. Stoddard, Rochester; (6) Legislation concerning tuberculosis: past, present and future, Hon. Horace White, Syracuse; (7) Taxation with relation to state care of consumptives,

Hon. Otto Kelsey, Geneseo; general discussion of these papers by John H. Pryor, Daniel Lewis, A. G. Root, S. B. Ward and others.

---

The Buffalo Academy of Medicine held meetings during the month of January, 1900, as follows:

*Section on Surgery.*—Tuesday evening, January 2d. Program: Discussion on prophylaxis and treatment of urethral stricture, by Wm. H. Heath, Wm. C. Phelps, J. B. Stoner, S. Y. Howell, M. Hartwig and others.

*Section on Ophthalmology.*—Monday evening, January 8th. Program: Old facts and new theories concerning eye strain, Lucien Howe. First paper—Historical and elementary. A case of tuberculous tumor of the nasal septum was presented by J. C. Clemesha.

*Section on Medicine.*—Tuesday, January 9th. Program: Typhlitis and scolecitis, A. L. Benedict. The treatment of acute appendicitis, DeLancey Rochester.

*Section on Pathology.*—Tuesday, January 16th. Lawson Tait, Stephen Y. Howell. Remarks by William Warren Potter.

*Section on Obstetrics.*—Tuesday, January 23d. Program: Differential diagnosis of disease of the appendix and Fallopian tubes, Dr. C. C. Frederick. Eruptive diseases of the female genitals, Dr. J. Henry Dowd.

---

The New York Physicians' Mutual Aid Association held its thirty-first annual meeting at the Academy of Medicine, 17 West 43d Street, Thursday, January 18, 1900, at 4 o'clock p. m. The following order of business was observed: Report of the trustees, election of officers. Action on proposed amendment to Section 1, Article IV., of the By-laws.

---

The annual meeting of the New York Academy of Medicine, was held at the Academy, No. 17 West Forty-third Street, Thursday evening, January 4, 1900, at which Dr. Reginald H. Sayre, treasurer of the board of trustees, reported that the last item on the Academy's list of indebtedness had been paid, and that the organisation begins the year free from debt, for the first time since the building of its home. Dr. Sayre said that the debt amounted to $2,000, and was the last of the building indebtedness. The structure cost about

$240,000. During the meeting the following new officers were installed. Vice-president, G. L. Peabody; trustee, Abraham Jacobi; treasurer of the board of trustees, Reginald H. Sayre, and treasurer of the Academy, H. L. Collyer.

At the conclusion of the business meeting the following papers were read: Pathology of migraine, Colman W. Cutler; Significance of migraine in Graves's disease, by the president, W. H. Thomson. The papers were then discussed by several of the members present.

---

THE Physicians' Club met at the home of Dr. J. Henry Dowd, 288 Franklin Street, Wednesday, January 10, 1900. The newly elected officers of the club were installed and an essay on Antiseptics in obstetrics was read by J. J. Walsh. The officers installed were: President, E. M. Dooley; vice-president, W. G. Ring; secretary, A. G. Bennett; council, Drs. J. J. Finerty, J. S. Porter, J. W. Nash, F. Boyle and C. E. Congdon.

